# Supplementary material for: Cis-regulatory fragments from the dissatisfaction gene identify novel mating behavior neurons in female Drosophila
Source: G3 (Bethesda). 2025 Oct 28;16(1):jkaf249. doi: 10.1093/g3journal/jkaf249 (PMC12774584; doi:10.1093/g3journal/jkaf249)
Supplement: jkaf249_Supplementary_Data [file jkaf249_supplementary_data.zip › Supplemental_Legends_G3-2025-406172.pdf]

Supplementary Figure 1. *cis*-regulatory fragments from the *dsf* gene label subsets of *dsf*-expressing neurons in the adult CNS. The intersection of *dsf*$^{\mathrm{Gal4}}$ and each *Dsf_CRE-LexA::p65* transgene targets various subsets of *dsf*-expressing neurons in the brain and ventral nerve cord of adult females and males. GFP-expressing neurons and DNCad (neuropil) are shown in black and light gray, respectively. Scale bar = 50 μm.

Supplementary Figure 2. *cis*-regulatory fragments from the *dsf* gene label subsets of *dsf*-expressing neurons in the larval CNS. The intersection of *dsf*$^{\mathrm{Gal4}}$ and each *Dsf_CRE-LexA::p65* transgene targets various subsets of *dsf*-expressing neurons in the brain and ventral nerve cord of larval females and males. GFP-expressing neurons and DNCad (neuropil) are shown in black and light gray, respectively. Scale bar = 50 μm.

Supplementary Video 1. Optogenetic activation of *dsf*$^{\mathrm{Gal4}}$ ∩ *Dsf_CRE_4-LexA::p65* > *CsChrimson::mVenus* unmated females. Upon photoactivation, a *dsf*$^{\mathrm{Gal4}}$ ∩ *Dsf_CRE_4-LexA::p65* > *CsChrimson::mVenus* unmated female opens her vaginal plates.

Supplementary Video 2. Optogenetic activation of *dsf*$^{\mathrm{Gal4}}$ ∩ *Dsf_CRE_4-LexA::p65* > *CsChrimson::mVenus* mated females. Upon photoactivation, a *dsf*$^{\mathrm{Gal4}}$ ∩ *Dsf_CRE_4-LexA::p65* > *CsChrimson::mVenus* mated female opens her vaginal plates, similar to an unmated female.

Supplementary Video 3. Optogenetic activation of *Dsf_CRE_4-LexA::p65* ∩ *dsf*$^{\mathrm{p65AD::Zp}}$ ∩ *VT026005-Zp::GDBD* > *CsChrimson::mVenus* unmated females. Upon photoactivation, a *Dsf_CRE_4-LexA::p65* ∩ *dsf*$^{\mathrm{p65AD::Zp}}$ ∩ *VT026005-Zp::GDBD* > *CsChrimson::mVenus* unmated female opens her vaginal plates.
