# Supplementary material for: Cis-regulatory fragments from the dissatisfaction gene identify novel mating behavior neurons in female Drosophila
Source: G3 (Bethesda). 2025 Oct 28;16(1):jkaf249. doi: 10.1093/g3journal/jkaf249 (PMC12774584; doi:10.1093/g3journal/jkaf249)
Supplement: jkaf249_Supplementary_Data [file jkaf249_supplementary_data.zip › Supplementary_Figure_2_G3-2025-406172.pdf]

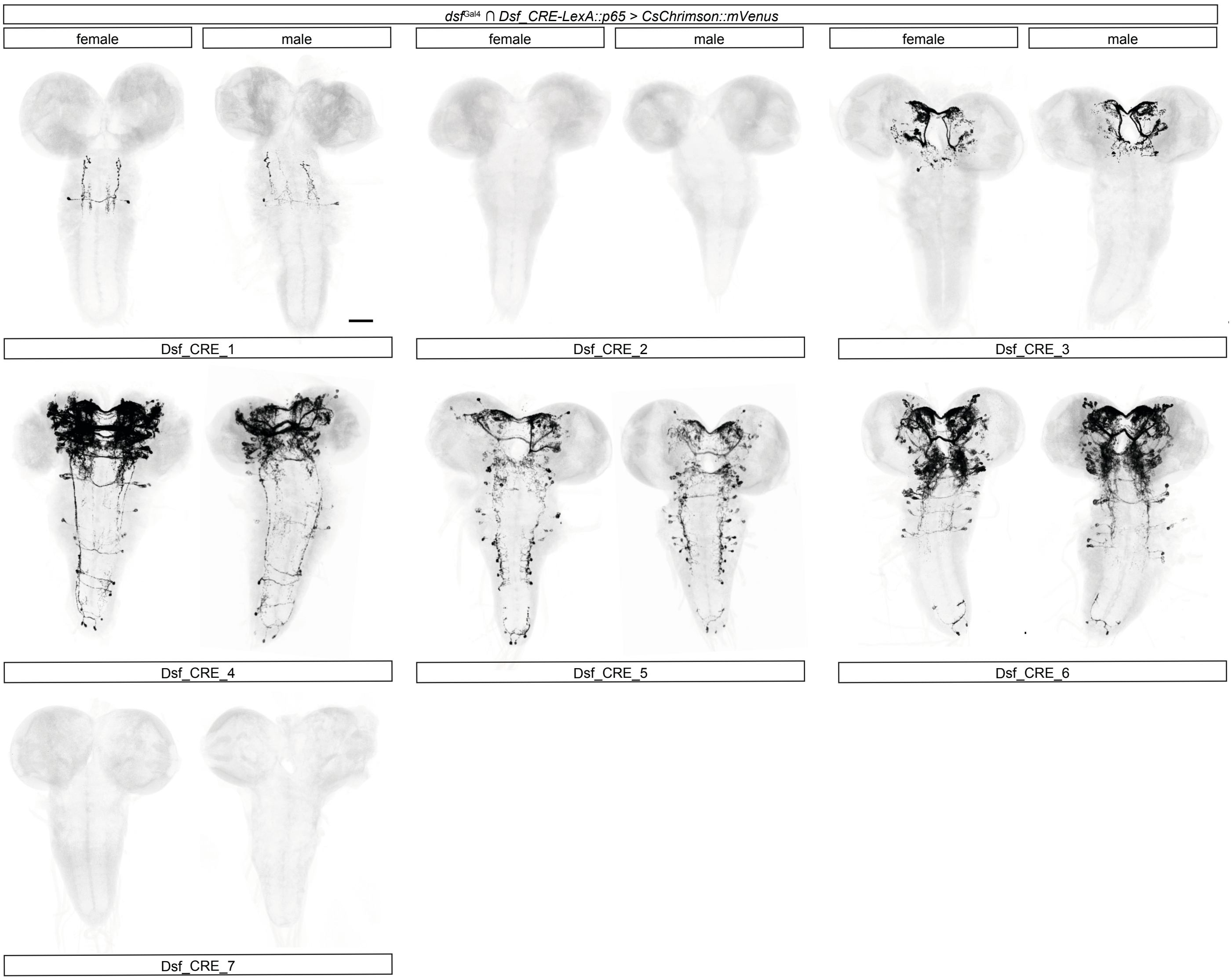

Supplementary Figure 2. cis-regulatory fragments from the dsf gene label subsets of dsf-expressing neurons in the larval CNS. The intersection of dsfGal4 and each Dsf_CRE-LexA::p65 transgene targets various subsets of dsf-expressing neurons in the brain and ventral nerve cord of larval females and males. GFP-expressing neurons and DNCad (neuropil) are shown in black and light gray, respectively. Scale bar = 50 m.
